# Feasibility of Telesimulation and Google Glass for Mass Casualty Triage Education and Training

**DOI:** 10.5811/westjem.2019.3.40805

**Published:** 2019-04-26

**Authors:** C. Eric McCoy, Rola Alrabah, Warren Weichmann, Mark I. Langdorf, Cameron Ricks, Bharath Chakravarthy, Craig Anderson, Shahram Lotfipour

**Affiliations:** * University of California, Irvine School of Medicine, Department of Emergency Medicine, Irvine, California; † University of California, Irvine School of Medicine, Department of Anesthesiology, Irvine, California; ‡ King Abdullah Bin Abdulaziz University Hospital, Department of Emergency Medicine, Riyadh, Saudi Arabia

## Abstract

**Introduction:**

Our goal was to evaluate the feasibility and effectiveness of using telesimulation to deliver an emergency medical services (EMS) course on mass casualty incident (MCI) training to healthcare providers overseas.

**Methods:**

We conducted a feasibility study to establish the process for successful delivery of educational content to learners overseas via telesimulation over a five-month period. Participants were registrants in an EMS course on MCI triage broadcast from University of California, Irvine Medical Simulation Center. The intervention was a Simple Triage and Rapid Treatment (START) course. The primary outcome was successful implementation of the course via telesimulation. The secondary outcome was an assessment of participant thoughts, feelings, and attitudes via a qualitative survey. We also sought to obtain quantitative data that would allow for the assessment of triage accuracy. Descriptive statistics were used to express the percentage of participants with favorable responses to survey questions.

**Results:**

All 32 participants enrolled in the course provided a favorable response to all questions on the survey regarding their thoughts, feelings, and attitudes toward learning via telesimulation with wearable/mobile technology. Key barriers and challenges identified included dependability of Internet connection, choosing appropriate software platforms to deliver content, and intercontinental time difference considerations. The protocol detailed in this study demonstrated the successful implementation and feasibility of providing education and training to learners at an off-site location.

**Conclusion:**

In this feasibility study, we were able to demonstrate the successful implementation of an intercontinental MCI triage course using telesimulation and wearable/mobile technology. Healthcare providers expressed a positive favorability toward learning MCI triage via telesimulation. We were also able to establish a process to obtain quantitative data that would allow for the calculation of triage accuracy for further experimental study designs.

## INTRODUCTION

Acts of terrorism, mass casualty incidents (MCI), and natural disasters continue to occur and overwhelm medical systems nationally and globally.^1^–^3^ An MCI is any incident where emergency medical services (EMS) resources are overwhelmed by the number and severity of casualties. Terrorism, both domestic and global, has recently been increasing. Specifically, the number of active shooter events and bombings has risen rapidly in the United States, with more than a third of events since 2000 occurring in the last three years.^4^–^5^ This increasing frequency has required healthcare providers to become more familiar with how to respond to these public health threats and emergencies.

Historically, the educational methods for these topics have included standard didactics and tabletop exercises (a low-fidelity type of simulation). However, passive educational delivery methods such as didactic lecture have been associated with the lowest average knowledge-retention rates compared to more active methods of learning.^6^–^7^ Simulation has been effectively used to train providers in areas such as EMS and critical care and provides a way to improve the quality of education and training to respond to these public health emergencies.

Simulation encompasses any process or technology that recreates a contextual background allowing the learner to make decisions, experience success, make mistakes, receive feedback, and gain confidence in an environment that is void of patient risk.^8^ Research has suggested that simulation is superior to conventional educational delivery methods in emergency and critical care medicine.^9^–^12^ However, in many areas around the world where healthcare providers must respond to acts of terror, there is a paucity or absence of simulation resources. Telesimulation is an innovative educational delivery method that can address this need.

Telesimulation is a process by which telecommunication and simulation resources are used to provide education, training, and/or assessment to learners at an off-site location.^13^ Because telesimulation is a new niche within simulation, the evidence demonstrating its effectiveness is scant. To our knowledge, no studies exist that evaluate the feasibility of telesimulation to deliver an EMS-based course to healthcare providers across continents. The objective of this study was to evaluate the feasibility and effectiveness of using telesimulation to deliver an EMS-based course on MCI training to healthcare providers on a different continent.

## METHODS

### Study Design and Setting

We performed a feasibility study to establish the process for successful delivery of educational content through a telesimulation course, that would yield data amenable for qualitative and quantitative analysis. The study was conducted over a five-month period with content creation, delivery, and broadcast from the University of California, Irvine Medical Simulation Center, a 65,000 square-foot state-of-the-art medical education center that provides telemedicine and simulation-based educational programs and continuing medical education courses for thousands of healthcare providers each year.^14^ Resources for education and training include a full-scale operating room, an inpatient ward room, emergency department resuscitation bay, obstetrical suite, and a critical care unit. The simulation center has a complement of full-time staff, including full-time simulation specialists.

**Population Health Research Capsule**

What do we already know about this issue?

*The increasing frequency of global terrorism has required healthcare providers to become familiar with how to respond to these public health threats and emergencies*.

What was the research question?

Can telesimulation be used to deliver a mass casualty incident training course to healthcare providers overseas?

What was the major finding of the study?

*We demonstrated the successful implementation of an intercontinental mass casualty incident triage course using telesimulation and wearable/mobile technology*.

How does this improve population health?

*Leveraging technology to improve knowledge and skills at the provider level allows for the optimization of healthcare delivery at the population level*.

### Selection of Participants

Participation in the study was obtained from registrants in an EMS-based course in MCI triage that was designed for this study and offered in collaboration with King Abdullah Bin Abdulaziz University Hospital and Princess Nourah bint Abdulrahman University Simulation and Skills Development Center in Riyadh, Saudi Arabia.

The study was open to all healthcare providers with a focus on emergency medical technicians, paramedics, nurses and physicians. The sole exclusion criterion was the inability to understand and speak the English language as the course and content materials were in English. The study was approved by the university’s institutional review board, and subjects provided informed consent.

### Interventions

The educational intervention in this study included an EMS-based course on MCI training on Simple Triage and Rapid Treatment (START) in the prehospital care setting. Mass casualty triage occurs when there is more than one casualty and the available resources require a provider to initiate care for one patient over another.^15^ The START system, developed by Hoag Hospital and the Newport Beach Fire Department (Newport Beach, California), helps prepare emergency personnel to quickly organize their resources to handle multi-casualty emergencies.^16^ It is designed to allow the provider to triage each patient in less than 60 seconds. This knowledge base and skill set is particularly critical for healthcare professionals responding to MCIs including active shooters and explosive devices. Currently, START remains the most commonly used mass casualty triage algorithm in the U.S.^17^

### Methods and Measurements

**Figure 1:**
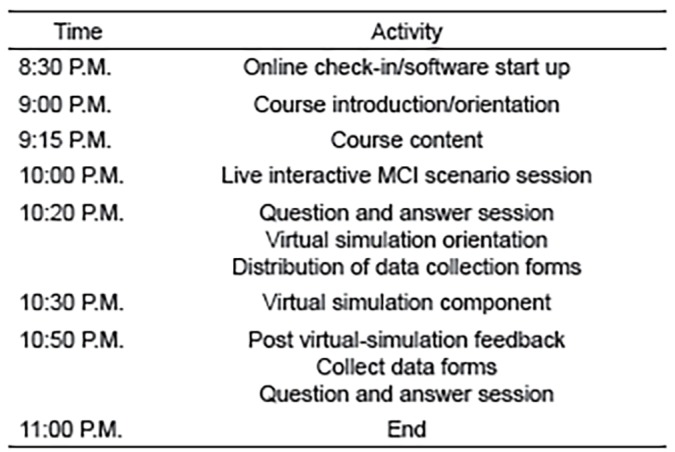
Timeline for telesimulation course from United States-based broadcasting institution. *MCI*, mass casualty incident.

The MCI course content was delivered using various telecommunication software resources. The course introduction and orientation was performed using join.me (https://www.join.me). Join.me is a web-based collaboration software application for screen sharing and online meetings. The course content was delivered using this software application as it allows real-time teleconferencing with simultaneous educational content broadcast. Educational content materials were delivered via PowerPoint (Version 12.0, Microsoft Corporation, Redmond, Washington). The course content was delivered by physicians who were board certified in both emergency medicine and EMS, and experienced in simulation course design, creation, and implementation. The course was delivered over 2.5 hours (which included a half-hour online check in and pre-course software testing period) (Figure 1).

After core content delivery of MCI education pertaining to START triage, the students underwent a live interactive training session to apply the knowledge gained. The course core content, live interactive training session, virtual simulation component, and question/answer sessions occurred over a two-hour time period (Figure 1). A MCI scenario was created and staged at the broadcasting institution simulation center using a combination of live standardized patients and high-fidelity simulation mannequins. We used the software platform EyeSight (Pristine Eyesight, Austin, Texas) for the live interactive MCI practical application training session. EyeSight allows real-time audio and video collaboration via smart glasses (Google Glass, Mountain View, California) and mobile devices (Figure 2A).

During this live interactive scenario, the course instructor played the role of a paramedic walking through a MCI scenario evaluating each patient and verbalizing information needed for participants to assign each patient the appropriate triage category. Course instructors at the receiving institution were available to answer any questions during the session (Figure 2B). After the live scenario was complete, a debriefing walkthrough of each patient ensued with the course instructors reviewing the appropriate assessment and triage categorization of each patient. The START triage method results in the assignment of patients into one of four categories: black (expectant), red (immediate), yellow (delayed), and green (minor).

The virtual simulation component of the course followed the live interactive MCI practical application training session. The virtual simulation consisted of a MCI of an active shooter in an office building. The virtual simulation was created, staged and recorded in an actual high-rise office building using standardized patients and moulage to create realistic-looking wounds. The simulation was recorded in the first-person perspective of an individual performing a continuous walkthrough of the MCI scene using Google Glass (Figure 2C). Each standardized patient underwent a pre-course training session regarding their roles and setting and also followed a pre-written script for his or her specific role. This pre-recorded MCI scenario served as the standardized resource that was used for individual participant assessment. Each student viewed the Google Glass recording of the high-rise active shooter incident and was responsible for categorizing each victim using START triage.

### Outcomes

The primary outcome of this feasibility study was to demonstrate the successful implementation of an EMS-based educational course on MCI management to healthcare providers in a different country via telesimulation. Designing, implementing, and reporting upon the process to deliver educational content to healthcare providers overseas was the major goal of this project. We also sought to obtain data amenable for both qualitative and quantitative analysis. The qualitative component of this study consisted of a survey to evaluate the learners’ thoughts, feelings, and attitudes about taking an EMS-based course in MCI triage via telesimulation. We used the quantitative data obtained to demonstrate the process and feasibility of collecting data to evaluate the diagnostic accuracy of triage performed by the learners in a course delivered via telesimulation.

### Analysis

We used descriptive statistics to express the percentage of participants with favorable responses to survey questions. Survey response categories consisted of ordinal data using a five-point Likert scale ranging from “strongly disagree” to “strongly agree.” Triage accuracy was summarized as percent correct.

## RESULTS

A total of 32 participants enrolled in the course: 12 physicians (37%); four nurses (13%); and five EMT/paramedics (16%). As the course offerings were open to all participants who could be responsible for providing service in a MCI, participants with varying backgrounds also enrolled, including two pharmacists (6%), and eight in “other” category (25%), which included educators, administrators and technicians. One participant (other*) did not fill out his or her profession on the survey.

**Figure 3:**
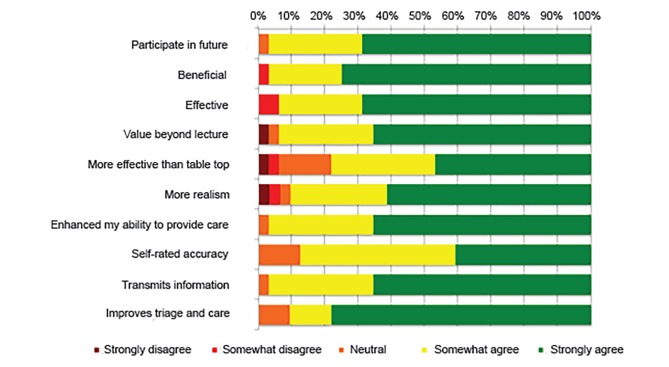
Survey results according to all response categories.

We evaluated feasibility by the successful utilization of resources for specific goals and in delivering the final product to the learners – an educational course on the EMS-based topic of MCI triage. Course participants’ thoughts, feelings, and attitudes toward learning EMS-based content on MCI triage were obtained by an immediate post-course survey that maintained subject anonymity. Learners reported that this experience added educational value beyond learning from standard lectures and that this method of virtual simulation is more effective than standard tabletop exercises to learn the MCI triage method (Figure 3). The participants also supported the notion that wearable technology can be an effective tool to transmit critical patient information in the prehospital care setting between providers. Furthermore, they reported that the telesimulation course enhanced their ability to provide care for patients involved in a MCI (Figure 3). Participants provided a favorable response to all questions on the survey regarding their thoughts, feelings, and attitudes toward learning EMS-based content via telesimulation.

For triage accuracy, we were able to collect data in real time of participants’ assessment of patients during the virtual simulation component of the course. These data allow the calculation of triage accuracy according to provider category and experience. As this feasibility study was not intended or designed as an observational-analytical study to evaluate the triage accuracy of participants, data are presented as simple descriptive statistics for illustrative purposes, without any inferences being stated or implied about the larger population from which the sample was drawn (Table 1).

## DISCUSSION

In this feasibility study we demonstrated the successful implementation of an EMS-based educational course on MCI triage training to healthcare providers overseas via telesimulation. This process allowed the collection of data amenable for both qualitative and quantitative analysis. As telesimulation is a relatively new niche, there is a paucity of literature to support the evidence base behind its use.

Our survey results revealed an overall positive view toward learning EMS content of MCI triage with telesimulation. Participants reported that this teaching experience added educational value above and beyond their learning from standard lectures and that this method of virtual simulation was more effective than standard tabletop exercises to learn the MCI triage method. The participants also supported the notion that wearable technology can be used as an effective tool to transmit critical patient information in the prehospital care setting between providers and reported that the telesimulation course enhanced their ability to provide care for patients involved in a MCI. Research has provided evidence to suggest that simulation is superior to conventional educational delivery methods in emergency and critical care medicine education.^9^–^12^ Our learner-favorability responses are consistent with those found in the telesimulation and simulation literature and support the research suggesting that simulation is superior to conventional educational-delivery methods.

The positive thoughts, feelings, and attitudes expressed by our learners toward telesimulation supports the findings in previous telesimulation studies. In a study using telesimulation to provide distance medical education, the authors used telesimulation to teach emergency scenarios with a remote expert providing instruction.^18^ A Likert-scale questionnaire revealed overwhelming satisfaction with the simulation-based distance training, and the authors concluded that simulation-based distance medical training proved to be a highly effective tool in improving emergency medical skills of junior physician trainees. They also reported that international simulation-based training may ultimately prove the most realistic platform for large-scale training of emergency medical personnel in less-developed countries and in rural/remote regions of the globe.

A military study designed to assess the efficacy and feasibility of training isolated emergency medical personnel at a naval hospital concluded that human patient simulation improves perceived preparedness and self-efficacy in U.S. Navy emergency medical personnel.^19^ They also reported that simulation and distance education allows isolated medical personnel the opportunity to practice skills unconstrained by time or distance.^19^ Telesimulation has also received favorable reviews in fields such as pediatric critical care and neonatal resuscitation.^20^–^22^

For the quantitative data collection component of the study, we were able to capture real-time learner assessment data during the virtual simulation component of the course. Using Google Glass to record the triage scenario used to assess participant triage accuracy provided a standardized experience for each learner from the first-person perspective. This immersive method of education and training is in stark contrast to abstract learning experiences such as standard didactics or tabletop exercises, which lack the virtual reality, stressful depiction, moulaged victims, high-stakes responsibility, personal perceived danger, and time pressure of a simulated first-person immersive experience. Research has shown that learning during emotional stress is associated with enhanced declarative memory for emotionally arousing events.^23^ The time learners had to evaluate each patient and make a decision with regard to their triage category – less than 60 seconds per patient – was consistent with that intended by the triage decision tool.

We were also able to demonstrate the successful implementation of wearable technology in the creation and delivery of an intercontinental MCI training course. Google Glass is a hands-free, wearable device that allows healthcare providers to evaluate and manage patients while simultaneously recording or transmitting data. The utility of wearable technology in healthcare has been evaluated in medical specialties including surgery,^24^–^26^ cardiology,^27^,^28^ ophthalmology,^29^ and emergency medicine.^30^,^31^ Our study contributes to the scientific knowledge pertaining to the utility of wearable technology in healthcare in the field of EMS and emergency medicine.

EMS is a relatively new subspecialty in emergency medicine, recognized by the American Board of Emergency Medicine (ABEM) in 2010, with the first certification exam in 2013.^32^ Literature regarding telesimulation in EMS is virtually nonexistent, and as a result there is limited research to support its evidence base.

Two previous reports using Google Glass have been described, both with substantial methodology limitations. In a feasibility study to determine the effect telemedicine has on the accuracy and timeliness to perform triage in an airplane-related MCI, the authors reported that there was no increase in triage accuracy when paramedics evaluated victims using Google Glass.^33^ They also reported that telemedicine required more time than conventional triage. Although reported as a feasibility study, the authors measured and reported quantitative metrics similar to that reported in an observational-analytical study with a nonrandomized intervention and control group. The small sample size (total of four paramedics), lack of randomization, different number of patients triaged by the teams, and unintended technology challenges that precluded real-time transmission of audio and video data through Google Glass, are significant limitations. The second related study used Google Glass during a full-scale exercise to perform visually guided triage and to identify casualties and collect georeferenced notes, photos, and videos into the debriefing.^34^ The authors reported that Google Glass is a promising technology both for telemedicine applications and augmented-reality disaster response support to increase operators’ performance, helping them to make better choices in the field.^34^

The aforementioned studies are similar to this report, in that Google Glass was used as an evaluative tool to collect data for assessment and/or educational purposes. This study differs by virtue of its use of wearable/mobile technology to provide *real-time* training in a live, simulated MCI with integrated debriefing with participant interaction. The wearable technology was also used to create the evaluation resource that was used during the virtual simulation component of the course. To our knowledge, this is the first study of its kind to implement an intercontinental MCI triage course to healthcare providers via telesimulation with Google Glass.

We also considered or encountered barriers and challenges when implementing this telesimulation course. We define barriers as those minimum requirements that must be attained or resources that must be obtained to conduct a telesimulation course. The major barriers include limited availability of telecommunication equipment, simulation resources, and personnel experienced in designing and delivering simulation-based course content (Table 2). Challenges pertain to those problems that educators may encounter in the interim between securing the minimum resources to conduct a telesimulation course and successfully delivering on the educational course protocol. The challenges we encountered were primarily operational. Choosing a software program proved to be an initial challenge as the country we were broadcasting to had strict limitations on the type of software platforms that could be used. Other challenges included Internet connectivity and live broadcasting to an institution 10 hours ahead in time.

## LIMITATIONS

The course delivered in this feasibility study was geared toward healthcare providers who would be responsible for managing patients in a MCI. Enrollment for the course was voluntary; thus, the sample may not be representative of all the professions that may be responsible for patient care during a MCI. However, we also believe that those responsible for managing patients during a MCI would be more enthusiastic to engage in this type of course.

The study was not designed as an observational-analytical study, nor was it powered to detect any specific significant difference between the groups. Having noted this, our results are consistent with triage performance demonstrated by healthcare providers undergoing START triage training using non-immersive educational methods.^35^,^36^ We believe this feasibility study makes possible future interventional studies to test whether specific content can be taught effectively with a new educational delivery method. And finally, the course was intended primarily for healthcare providers. However, professionals in the educational setting also participated. The background of this group (“other” category) was heterogeneous and their summary performance measure may not be representative of any particular individual profession in that group.

This telesimulation obviated the need for expert instructors from the sending site to travel to the recipient site to deliver the content, saving cost. Nevertheless, substantial infrastructure investments were necessary on both ends to make the telesimulation possible.

## CONCLUSION

This study demonstrates that implementation of an intercontinental MCI-triage training course, and the use of wearable/mobile technology to create and deliver the content though telesimulation, was feasible and well-accepted by learners. Further study is needed to validate telesimulation as a content delivery method to emergency providers.

## Figures and Tables

**Figure 2A:**
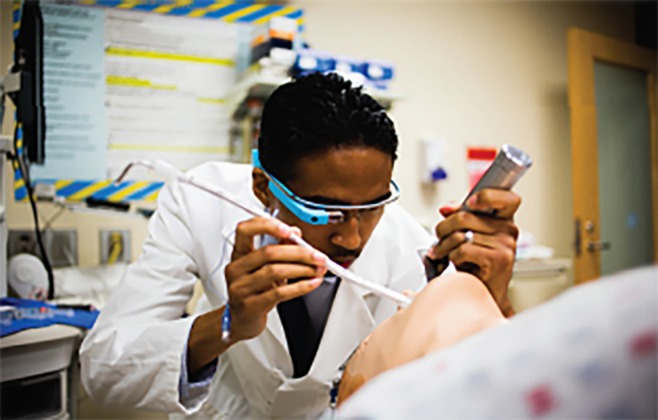
Healthcare provider using wearable technology (Google Glass) while performing advanced airway procedure.

**Figure 2B:**
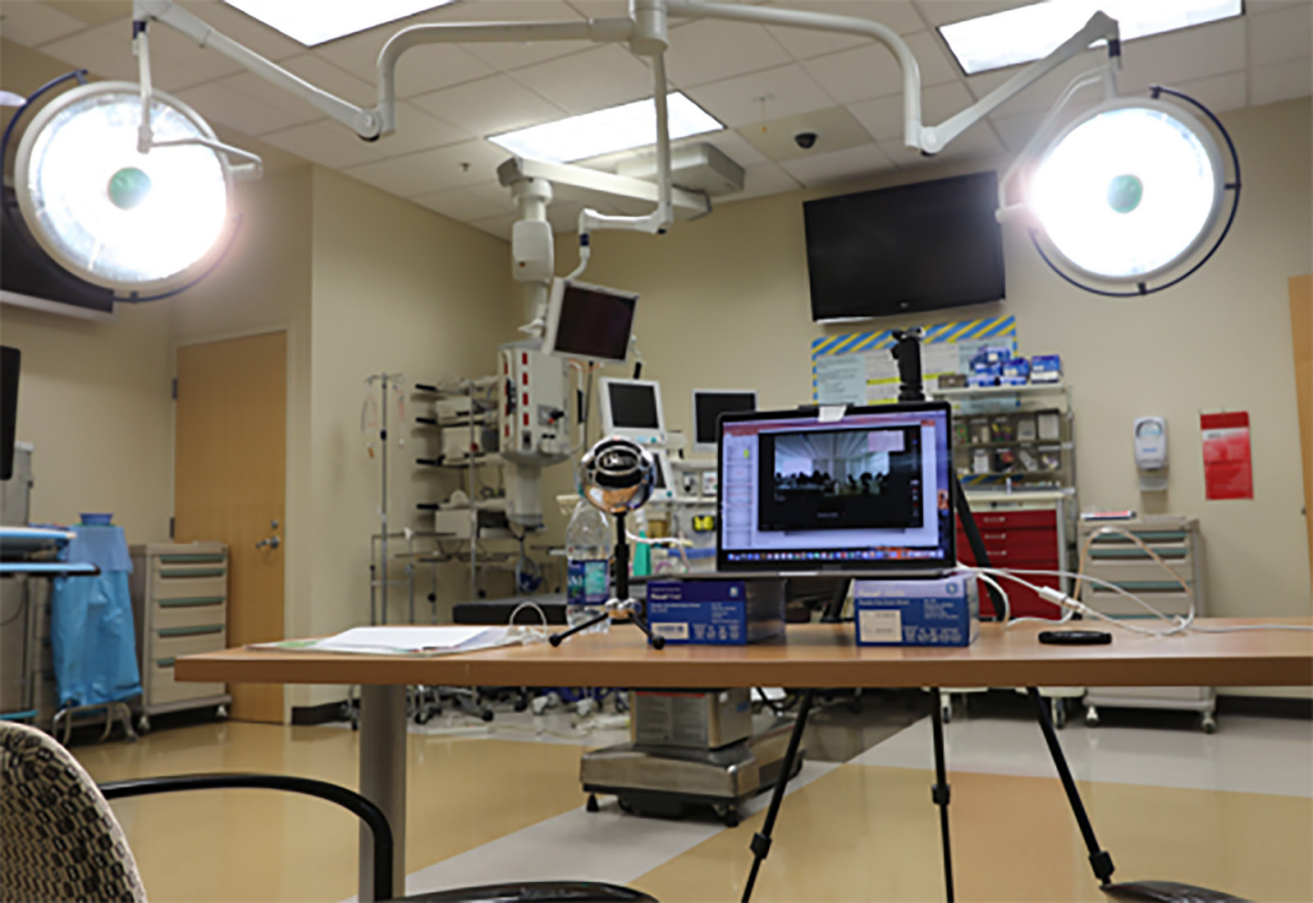
Broadcasting institution location with laptop showing live feed to classroom at receiving location. Instructors at broadcasting institution were able to communicate live to students and instructors overseas via Google Glass, laptop, desktop, and wall-mounted TV monitors.

**Figure 2C:**
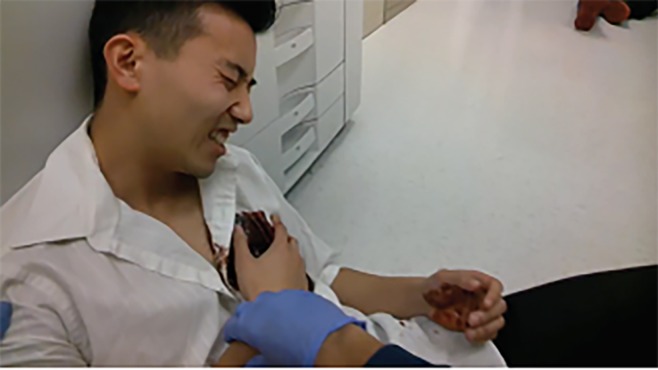
Photograph depicting first-person view frame captured from Google Glass during the virtual simulation component scenario of a mass casualty incident training course. Students at the receiving institution experienced real-time, first-person perspective triage after a staged, active-shooter scenario.

**Table 1:** Diagnostic triage accuracy according to profession.

Group	N	% correct triage
Physicians	12	84
Nurses	4	88
EMT/Paramedics	5	60
Pharmacists	2	85
Educator/technician/other	8	78

*EMT*, emergency medical technician.

n=sample size; other=educator, technician, administration.

**Table 2:** Barriers and challenges to telesimulation course implementation.

Barriers
Acquiring telecommunication resources
Acquiring simulation resources
Securing subject matter expert(s)
Securing educators experienced in simulation
Financing
Challenges
Internet connectivity
Choosing appropriate multimedia software
Familiarity with technology
Course scheduling (for different time zones)
Establishing inter-institutional relationships
